# Editorial: The Role of the Distinctions between Identification/Production and Perceptual/Conceptual Processes in Implicit Memory: Findings from Cognitive Psychology, Neuroscience and Neuropsychology

**DOI:** 10.3389/fpsyg.2017.01129

**Published:** 2017-06-30

**Authors:** Matthew W. Prull, Pietro Spataro

**Affiliations:** ^1^Department of Psychology, Whitman CollegeWalla Walla, WA, United States; ^2^Department of Psychology, Sapienza University of RomeRome, Italy

**Keywords:** implicit memory, priming, repetition priming, involuntary memory, attention

The present collection of papers represents recent work on repetition priming. In a standard repetition priming experiment, participants first encode materials such as words or pictures into memory. Later, they are asked to process those materials together with new, unencoded materials of a similar nature in a task that makes no reference to earlier encoding. For example, after studying a word such as *grape*, the probability of using *grape* as a response to the corresponding word stem (GRA_____) is enhanced under instructions to complete the stem with the first word that comes to mind. Moreover, relative to unencoded words, participants are more successful at identifying encoded words when presented at perceptual threshold, and faster at verifying that encoded words are exemplars of superordinate categories.

What made priming so exciting to researchers was the fact that the principles governing priming often differed from the principles that governed explicit memory. For example, read/generate manipulations had opposite effects on cued recall tests of explicit memory and threshold identification measures of priming, encoding-phase divided attention had deleterious effects on recall and recognition tests but had little or no effect on priming tasks such as lexical decision and category verification, and memory impairments that accompanied aging, brain injury, and disease often dissociated explicit memory and priming in various ways (Mulligan and Besken, 2013). These observations forced researchers to develop new theoretical frameworks that could accommodate the findings.

The following collection of papers was inspired by one such framework, the identification/production framework (Vaidya et al., 1997; Gabrieli et al., 1999; Prull, 2004, 2010; Geraci, 2006; Marques et al., 2016). In that framework, one type of priming consists of identification tasks in which stimuli must be identified, classified, or verified, and retrieval processes converge on one unique response. Category verification is an example of an identification task because the task requires identifying the superordinate category to which a word belongs. In contrast, production priming tasks are those in which a test cue initially guides retrieval toward many potential responses from which one must choose. Word stem completion is an example of a production priming task because usually several words can complete a given stem, and participants must select one response from among the various competing alternatives activated by the stem cue. Not all tasks are easily classified, however, and researchers have disagreed over whether certain tasks should be considered production or identification tasks (Spataro et al., 2017). Nevertheless, the distinction was useful because it helped to explain varieties of priming in healthy and memory-impaired populations. Thus, priming on several production tasks appeared to reflect mnemonic processes supported by anterior brain regions that were sensitive to Alzheimer's disease pathology (AD) and encoding-phase division of attention (DA). In contrast, priming on identification tasks appeared to reflect memory processes supported by posterior brain regions that were affected less by AD and DA (Gabrieli et al., 1999; Fleischman et al., 2001).

Divided attention was a key factor that initially motivated the framework, and several papers in the present collection continue in that tradition by investigating the attentional requirements of identification and production forms of priming. Gomes and Mayes found that a selective attention manipulation at encoding that reduced explicit memory did not reduce priming for objects and non-objects in an object-decision task. However, Ballesteros and Mayas found significant reductions of priming on a semantic classification task when attention was diverted from targets at the time of encoding. These identification tasks are usually insensitive to attentional manipulations at encoding, so clearly more investigation is in order. In contrast, Prull et al. examined the attentional demands of priming in category exemplar generation, a production priming task, at the time of retrieval. Building on earlier studies, those researchers found that priming was insensitive to divided attention at retrieval even when the secondary or distracting task required similar conceptual processes as those required by the implicit memory task itself. The retrieval of information in production priming tasks therefore appears to be automatic.

Other researchers focused on potential mechanisms that drive priming in specific tasks. Soler et al. focused on word stem completion, a production priming task, by examining the impact of various linguistic factors (e.g., word frequency, familiarity), as well as the number of possible solutions, on word-stem priming. Of interest is their finding that, when word stems have unique solutions, priming behaves like other identification priming tasks in which there is only one possible response. Their results highlight the idea that response competition—prevalent in many production priming tasks—can be manipulated independently of the production or identification classification of the task. In a similar vein, Weatherford et al. explored factors that affect priming in an identification task—semantic classification—and reported different patterns of priming depending on features such as the semantic relatedness and the modality match of the studied material to target material. Together, these studies underscore the complexity of the mechanisms leading to priming on identification and production tasks.

Finally, two studies examined identification and production priming in relation to various participant characteristics. LaVoie et al. found that word-stem completion priming was greater among those with inconsistent handedness compared to consistent handedness. No such handedness difference was found for the identification task of perceptual identification. This finding suggests that interhemispheric transfer of information may be more critical for production priming than for identification priming. Ruch et al. examined priming for words presented during sleep, and reported that word-entrained up-states in the EEG patterns while sleeping correlated with the magnitude of priming in threshold identification and classification tasks. These findings are novel by demonstrating that the magnitude of identification priming can be predicted from brain activity during encoding.

To conclude, we hope that, by providing a comprehensive overview of recent developments concerning the identification/production framework of implicit memory, the present collection will stimulate additional research aimed at deepening our understanding of the cognitive and neural underpinnings of priming processes. Above all, we hope that you will enjoy reading these papers as much as we enjoyed assembling them for you.

## Author contributions

All authors listed have made a substantial, direct and intellectual contribution to the work, and approved it for publication.

### Conflict of interest statement

The authors declare that the research was conducted in the absence of any commercial or financial relationships that could be construed as a potential conflict of interest.
